# Longitudinal MRI-visible perivascular space (PVS) changes with long-duration spaceflight

**DOI:** 10.1038/s41598-022-11593-y

**Published:** 2022-05-05

**Authors:** Kathleen E. Hupfeld, Sutton B. Richmond, Heather R. McGregor, Daniel L. Schwartz, Madison N. Luther, Nichole E. Beltran, Igor S. Kofman, Yiri E. De Dios, Roy F. Riascos, Scott J. Wood, Jacob J. Bloomberg, Ajitkumar P. Mulavara, Lisa C. Silbert, Jeffrey J. Iliff, Rachael D. Seidler, Juan Piantino

**Affiliations:** 1 Department of Applied Physiology and Kinesiology, University of Florida, 1864 Stadium Rd., Gainesville, FL USA; 2 Layton-NIA Oregon Aging and Alzheimer’s Disease Research Center, Department of Neurology, Oregon Health and Science University, Portland, OR USA; 3 Advanced Imaging Research Center, Oregon Health and Science University, Portland, OR USA; 4 Division of Child Neurology, Department of Pediatrics, Doernbecher Children’s Hospital, Oregon Health and Science University, 707 SW Gaines St., CDRC-P, Portland, OR 97239 USA; 5 KBR, Houston, TX USA; 6 Department of Diagnostic and Interventional Imaging, University of Texas Health Science Center at Houston, Houston, TX USA; 7 NASA Johnson Space Center, Houston, TX USA; 8 Neurology, Veteran’s Affairs Portland Health Care System, Portland, OR USA; 9 Department of Psychiatry and Behavioral Sciences, University of Washington School of Medicine, Seattle, WA USA; 10 Department of Neurology, University of Washington School of Medicine, Seattle, WA USA; 11 VISN 20 Mental Illness Research, Education and Clinical Center (MIRECC), VA Puget Sound Health Care System, Seattle, WA USA; 12 Norman Fixel Institute for Neurological Diseases, University of Florida, Gainesville, FL USA

**Keywords:** Neuroscience, Physiology, Neurology

## Abstract

Humans are exposed to extreme environmental stressors during spaceflight and return with alterations in brain structure and shifts in intracranial fluids. To date, no studies have evaluated the effects of spaceflight on perivascular spaces (PVSs) within the brain, which are believed to facilitate fluid drainage and brain homeostasis. Here, we examined how the number and morphology of magnetic resonance imaging (MRI)-visible PVSs are affected by spaceflight, including prior spaceflight experience. Fifteen astronauts underwent six *T*_1_-weighted 3 T MRI scans, twice prior to launch and four times following their return to Earth after ~ 6-month missions to the International Space Station. White matter MRI-visible PVS number and morphology were calculated using an established, automated segmentation algorithm. We validated our automated segmentation algorithm by comparing algorithm PVS counts with those identified by two trained raters in 50 randomly selected slices from this cohort; the automated algorithm performed similarly to visual ratings (r(48) = 0.77, *p* < 0.001). In addition, we found high reliability for four of five PVS metrics across the two pre-flight time points and across the four control time points (ICC(3,*k*) > 0.50). Among the astronaut cohort, we found that novice astronauts showed an increase in total PVS volume from pre- to post-flight, whereas experienced crewmembers did not (*p* = 0.020), suggesting that experienced astronauts may exhibit holdover effects from prior spaceflight(s). Greater pre-flight PVS load was associated with more prior flight experience (r = 0.60–0.71), though these relationships did not reach statistical significance (*p* > 0.05). Pre- to post-flight changes in ventricular volume were not significantly associated with changes in PVS characteristics, and the presence of spaceflight associated neuro-ocular syndrome (SANS) was not associated with PVS number or morphology. Together, these findings demonstrate that PVSs can be consistently identified on *T*_1_-weighted MRI scans, and that spaceflight is associated with PVS changes. Specifically, prior spaceflight experience may be an important factor in determining PVS characteristics.

## Introduction

Spaceflight-induced physiological stressors have been well documented, including exposure to microgravity, ionizing radiation, and circadian disruption^1,2^. Astronauts returning to Earth after spaceflight exhibit structural brain changes, including an upward shift of the brain within the skull^3–5^, regional gray matter volume changes^4–7^, and altered white matter volume and microstructural integrity^6–8^. Spaceflight is also associated with alterations in cerebrospinal fluid (CSF) flow, evidenced by ventricular expansion^3,5–7,9–12^ and redistribution of CSF away from the top of the brain^3,6,8^. Clinically, approximately 33% of astronauts returning from long-duration spaceflight exhibit spaceflight associated neuro-ocular syndrome (SANS), a constellation of ocular structural changes including optic disc edema, choroidal folding, globe flattening, and hyperopic refractive error shifts^13,14^. The etiology of SANS remains unknown but could include fluid shifts, increased intracranial pressure, and/or altered CSF drainage with spaceflight^13^.

Brain CSF^3,5,8,9,12^, gray^4,5^, and white matter^8,12^ changes with spaceflight are associated with the number and duration of prior missions. Furthermore, some brain changes (particularly, lateral and third ventricular expansion) show only partial recovery at 6–12 months post-flight^5,6,10,11^; thus, astronauts who return to space may experience holdover effects from prior flights. For instance, our prior work^5^ reported that a shorter time between missions was associated with larger pre-flight lateral ventricles (even after correcting for age effects). Moreover, we found that experienced astronauts who exhibited *larger* pre-flight lateral ventricles also showed *less* lateral ventricular expansion in a subsequent flight compared to novice crewmembers^5^. Together, these findings highlight both frequency and duration of spaceflight as important factors for understanding how microgravity affects cranial fluid shifts and CSF drainage.

According to the classic model, CSF produced by the choroid plexus circulates to the subarachnoid space before exiting the brain via the arachnoid granulations. However, recent evidence suggests that CSF in the subarachnoid space also flows into the brain parenchyma through the periarterial spaces surrounding the penetrating arteries^15^. Once in the brain parenchyma, CSF exchanges with interstitial space fluid (ISF), eventually returning to the cisternal CSF along the perivenous pathway^16^. Interstitial and CSF solutes are cleared from the cranium in part through the cervical lymphatic system via dural lymphatic vessels located along the superior sagittal sinus, or near the cribriform plate^17^. This perivascular exchange of CSF and ISF along what has been termed the glymphatic pathway plays an important role in the removal of cerebral interstitial solutes and wastes^18,19^. Perivascular space (PVS) enlargement visible on MRI is proposed to represent a disruption of this process and has been observed in conditions associated with glymphatic dysfunction^20–25^.

To date, the effect of microgravity on perivascular CSF circulation remains unknown. We hypothesized that the upward brain shift resulting from microgravity exposure might compress the meningeal lymphatics running along the superior sagittal sinus, impairing CSF/ISF exchange, and/or impair CSF resorption in the arachnoid (Pacchioni) granulations. Disruption of fluid drainage would be evidenced by an increase in MRI-visible PVS number and/or size (i.e., enlargement of PVS volume, length, or width). In the present work, we examine PVS number and size in astronauts pre- and post-approximately 6-month-long International Space Station missions. Here we define PVS as the interstitial fluid-filled space surrounding the intracranial vessels in the brain parenchyma^26^, with a diametric measure of less than 3 mm^27^. Due to the high health standards set for astronauts, the MRI-visible loads exhibited by this population are likely PVSs and not manifestations of disease (i.e., small vessel disease) or brain injury. Although our original study design^28^ did not include PVS analysis, the literature that has emerged since this time^19,20,22,25,29,30^ led us to hypothesize that spaceflight would impact PVS number and morphology.

The objectives of this study included: (1) To define the effects of spaceflight on PVS characteristics. We predicted an increase in white matter PVS number, volume, length, and/or width after flight. (2) To explore the differences in PVS changes with flight for novice versus experienced astronauts. We predicted that novice astronauts would exhibit more changes in PVS number and size from pre- to post-flight. We also predicted that, within the experienced astronaut subgroup, there would be a correlation between a greater number of days previously spent in spaceflight and a higher number and larger size of PVSs at baseline. (3) To determine the relationship between ventricular expansion and PVS changes with spaceflight. We predicted that greater pre- to post-flight increases in the sum of lateral and third ventricular volume would be associated with an increase in PVS number and size. (4) To examine differences in PVS changes with flight for those who exhibited signs of SANS vs. those who did not (no-SANS). We predicted greater increases in PVS number and size from pre- to post-flight for the SANS versus no-SANS individuals.

## Methods

### Participants

This was a longitudinal cohort study. Fifteen astronauts and 16 ground-based controls provided their written informed consent to participate in this study. All flight participants completed the highly competitive astronaut selection process, which includes various health, fitness, and education requirements^2^. Astronauts were also screened for MRI contraindications prior to participation in this study. The ground-based control participants were employees at NASA Johnson Space Center who held at least a master’s degree in a science or engineering field and passed a minimum physical fitness standard (i.e., Air Force Class III equivalent physical examination). The University of Michigan, University of Florida, and NASA Institutional Review Boards approved all study procedures. All methods were performed in accordance with the relevant guidelines and regulations. This analysis was implemented as part of a larger NASA-funded project investigating brain^5,31^ and behavioral^32^ changes occurring with long-duration ISS missions^28^.

### Experimental design

The astronauts completed approximately 6-month International Space Station missions (average mission duration: 167 days; Table 1), as well as MRI scans at six time points: approximately 180 and 60 days prior to launch (i.e., Launch-180 and Launch-60 days), as well as approximately 4, 30, 90, and 180 days after return to Earth (i.e., Return + 4, Return + 30, Return + 90, and Return + 180 days, respectively). One astronaut withdrew from the study before their Return + 180 days session; thus, we acquired data from 14 of 15 astronauts at this time point. Of note, this withdrawal did not affect any statistical analyses, which focused on pre- to post-flight changes and relationships of pre-flight PVS metrics with prior flight experience.

**Table 1:** Astronaut and control group demographics and flight experience.

	Astronauts					Controls^e^		
	All Astronauts	Novice	Experienced	t or χ^2^	*p*	All Controls	t or χ^2^	*p*
**Demographics**								
*n*	15	9	6	–	–	11	–	–
Age (years)^a^	47.46 (6.28)	43.98 (4.90)	52.67 (4.18)	3.68	0.003**	42.28 (10.60)	1.44	0.169
Sex	27% F	33% F	17% F	0.51	0.475	27% F	0.001	0.973
**Current mission**								
Mission duration (days)	190.67 (57.36)	166.78 (31.37)	226.50 (71.21)	1.93	0.099	–	–	–
Time elapsed between landing and return + 4 days MRI scan	4.53 (1.13)	4.83 (0.41)	4.33 (1.41)	1.00	0.341	–	–	–
**Experienced astronauts (*n* = 6)**^b^								
Number of previous missions	–	–	2.00 (0.89)	–	–	–	–	–
Past flight days (days)^c^	–	–	187.50 (151.67)	–	–	–	–	–
Time elapsed since last flight (years)^d^	–	–	5.77 (1.60)	–	–	–	–	–

**p < 0.01. For each metric, we report the mean (standard deviation) for the whole astronaut cohort, the novice and experienced subgroups, and the whole control group. We also report the results of two-sample t-tests to characterize differences in demographic variables between the novice and experienced astronaut groups, and between the whole astronaut cohort and the whole control group. For sex, we report the percentage of females included in each group and the result of a Pearson’s Chi-square test for differences in the sex distribution within each group.

^a^Age represents age, in years, at the date of launch for the astronauts and age at the first MRI scan for the controls.

^b^Here, we report further details regarding previous spaceflight experience for *n* = 6 of 15 astronauts who completed at least one flight before the current ISS mission.

^c^Past flight days indicates the total number of previous days spent in flight, not including the current mission.

^d^Time elapsed since the last flight is the time from the landing day of the most recent flight to the launch of the current mission.

^e^Here we include only those control group participants with complete MRI datasets (*n* = 11). See “Methods” for further details.

Significant values are in bold.

The controls completed the same MRI testing at 4 time points across 3 months (i.e., baseline and at approximately 10, 50, and 90 days following the baseline testing). Two controls dropped out before the final time point, so *n* = 14 subjects with MRI scans at these time points. Due to budget and time constraints, data from these control individuals were collected with the intention for use as control data for multiple spaceflight and spaceflight analog studies. That is, the data provide an estimate of stability over a 3-month period. Thus, the control testing timeline and demographics were not matched to the astronauts, which limited our ability to directly compare the controls to the astronauts. Rather, we leveraged the ground-based control group as converging evidence that PVS changes identified within the astronauts were due to spaceflight and not simply a function of test–retest reliability. There was a partial overlap between the MRI scan dates for the controls and the astronauts. Moreover, since we collected astronaut data at two pre-flight time points, we were able to test for pre-flight stability of our PVS metrics and the astronauts could serve as their own statistical controls. We have used a similar study design in our previous spaceflight analog work^33,34^, and such a study design is common in the longitudinal neuroimaging literature more broadly (e.g.^35,36^).

### Structural MRI acquisition

All MRI scans were collected using the same 3.0 T Siemens Magnetom Verio MRI scanner using a 32-channel head coil at the University of Texas Medical Branch at Victory Lakes, TX. Recent evidence suggests that time of day can affect PVS metrics^37^; however, as all MRI scans for the current study were collected in the morning, our PVS measurements were likely not influenced by these timing effects. We collected a *T*_1_-weighted structural MRI scan using the following imaging parameters: magnetization-prepared rapid gradient-echo (MPRAGE) sequence, TR = 1.9 s, TE = 2.32 ms, TI = 900 ms, number of phase encoding steps = 255, phase oversampling = 0, slice oversampling = 18.2%, no parallel imaging, flip angle = 9°, FOV = 250 × 250 mm, slice thickness = 0.9 mm, 176 sagittal slices, matrix = 512 × 512, voxel size = 0.488 × 0.488 × 0.9 mm = 0.214 mm^3^.

### Measurement of ventricular volume

We first processed all *T*_1_-weighted scans using the Computational Anatomy Toolbox (CAT12.6, version 1450)^38,39^ implemented within Statistical Parametric Mapping 12 (SPM12; version 7219)^40^ using MATLAB (R2016a, MathWorks Inc., Natick, MA). We implemented standard CAT12 preprocessing steps to segment the images by tissue type and to extract total intracranial and total white matter volumes. In addition, we extracted native space total lateral and third ventricular volumes for each participant and time point, as defined by the Neuromorphometrics volume-based atlas map (http://Neuromorphometrics.com). We used the sum of the total lateral and third ventricular volumes in all statistical models because extensive prior work has demonstrated substantial changes in both lateral and third (but not fourth) ventricular volume with spaceflight^5–7,10,11^. We refer to this sum as “ventricular volume” throughout the remainder of the manuscript.

### Characterization of PVSs

#### PVS segmentation

White matter PVS identification was carried out using our previously-described algorithm that searches for hypointense structures that meet certain pre-specified morphological criteria^29,41,42^. Briefly, first, the 3D *T*_1_-weighted MRI images were skull-stripped using the Brain Extraction Tool (BET) from the FMRIB Software Library (FSL; Version 5.0). We then resliced the *T*_1_-weighted images and CAT12-produced white matter masks to 0.5 mm isotropic voxel size, dilated the white matter masks by two voxels, filled any holes, and, finally, eroded the corrected white matter masks by two voxels to avoid partial volume effects, as described by Promjunyakul et al.^43^. Each voxel was then subjected to a two-step morphological analysis as follows. First, a sphere with a 3 mm radius was constructed around each voxel within the eroded white matter mask, and the voxels within that sphere were ranked based on their intensity; each voxel was subsequently marked by its neighborhood rank. Another sphere with a 4 mm radius was constructed around each voxel. The average intensity difference between the voxel and the surrounding voxels was calculated and assigned to each voxel (*3dLocalstat*, AFNI). A voxel was considered for morphological analysis if: (a) the voxel resided in the eroded white matter mask; (b) the difference between the voxel’s intensity and the average intensity of the surrounding voxels was > 15%; (c) the voxel’s intensity fell in the bottom fifth percentile of its neighbors. Second, voxel clusters that were > 1 mm^3^ (3D corner-to-corner connectivity, *3dclust* type 3, AFNI) were considered for morphologic constraint analysis. For each cluster, we calculated linearity, width, length, and total volume in MATLAB. Clusters that were (a) < 16.41 mm wide and (b) had a linearity of at least 0.8 were considered putative PVS and were manually verified by a trained rater (ML). Any identified false alarms (8.9% of total putative PVS across both the astronauts and controls) were removed from the final statistical analyses, as done in our prior work^29,41^; false alarms were largely due to a partial voluming of other structures (e.g., ventricle and basal ganglia). We then used custom MATLAB code to calculate five key outcome metrics, as done in our past work^29,41,42^. These outcomes included two metrics that describe total PVS load: (1) total PVS volume (i.e., the sum of the volumes in mL of each distinct PVS in the white matter), and (2) total PVS number (i.e., the total number of distinct PVS in the white matter). The remaining three metrics were characteristics of each distinct PVS: (3) volume (mm^3^), (4) length (mm), and (5) width (mm). For these three metrics, we then calculated the median volume, median length, and median width across all PVS clusters for each participant at each time point.

#### Image quality control

Out of the 89 total astronaut scans (i.e., 6 time points × 15 astronauts, with one astronaut who withdrew before the final post-flight scan) and 62 total control scans (i.e., 4 time points × 16 controls, with two individuals who withdrew before the final scan), we excluded 2 astronaut scans and 8 control scans in total from further analysis of PVS metrics. These excluded scans were degraded by motion, noise, or other artifacts, as determined by visual inspection by trained raters (ML, DS, and JP). The excluded astronaut scans included one pre-flight scan (i.e., Launch-60 days); for this participant, we calculated the pre- to post-flight change in PVS metrics using their single baseline scan (i.e., Launch-180 days). The other excluded astronaut scan was from a later post-flight timepoint (i.e., from Return + 180 days). For the control scans, we excluded the participant entirely if one of their four scans was excluded due to artifact; this left us with *n* = 11 complete control datasets.

#### Validation of PVS segmentation algorithm

In order to validate our PVS segmentation algorithm, we compared the results from the PVS detection pipeline to visual PVS inspection by two experienced raters (ML and JP). Briefly, these two experienced raters counted the number of PVS in a single slice in 50 randomly selected MRIs from this cohort. The raters were blinded to the number of PVS generated by the algorithm. The average PVS count between the two raters was then compared to the PVS counts generated by the algorithm using Pearson correlation coefficient.

### Statistical analyses

We conducted all statistical analyses using R 4.0.0^44^. Of note, no mathematical correction was made for multiple comparisons in any of the described statistical analyses. Instead, in each table in the main text and supplement, we report all individual *p* values and confidence intervals^45,46^.

#### Reliability of PVS metrics

We first tested for stability in the five PVS metrics and ventricular volume by calculating the intraclass correlation coefficient (ICC) between the two pre-flight scans for the astronauts, and across all four scans for the control group. As in our previous work^33^, we planned a priori to exclude from further statistical analyses any metric with an ICC(3,*k*) value below 0.5 (moderate reliability^47^).

#### PVS changes with spaceflight

We used five separate multivariate linear models to test for changes with spaceflight in each PVS metric and ventricular volume. We entered the difference between the PVS metric or ventricular volume from the last pre-flight time point (Launch-60 days) to the first post-flight time point (Return + 4 days) as the outcome variable. We were specifically interested in whether the intercept was statistically significant (*p* < 0.05), thereby indicating a change in the PVS metric or ventricular volume with spaceflight.

Next, we tested whether any covariates affected pre- to post-flight change in PVS or ventricular volume metrics. We entered the following covariates into the full model: sex, mean-centered age at launch, mean-centered flight duration, mean-centered time elapsed from landing to the Return + 4 days MRI scan, and novice versus experienced status. We then used *stepAIC*^48^ to produce a final model that retained only the best predictor variables; *stepAIC* uses a stepwise approach to select a maximal model based on the combination of predictors that produces the smallest Akaike information criterion (AIC).

To account for individual differences in total brain tissue volumes and allow for direct inter-subject comparison of PVS metrics, in the respective models we divided each participant’s total PVS volume (mm^3^) and total PVS number by their average pre-flight total brain white matter volume (cm^3^). These corrections are identical to those applied in our previous PVS work^29,41^. To account for individual differences in head size and allow for direct inter-subject comparison of ventricular volume, in the respective model we expressed each astronaut’s ventricular volume divided by their average baseline total intracranial volume^5^. A correction for head size was not necessary for the median PVS metrics^29,41^. We ensured that each model met the relevant assumptions (i.e., independence, outliers, linearity, and normality). We tested for normality using visual inspection of qqplots and the Shapiro test (*p* > 0.05).

#### Relationship between pre-flight PVS characteristics and previous flight experience

We then conducted an exploratory analysis on the subset of *n* = 6 experienced astronauts. We used stepwise multivariate linear regression to examine whether average baseline PVS metrics or ventricular volume associated with the total number of previous days spent in space (i.e., total days in space, not including the current mission). We first ran a reduced model including previous flight days as the only predictor. If there was a significant (*p* < 0.05) linear relationship between previous flight days and the respective PVS metric, we then planned to calculate a full model including sex and mean-centered age at launch as additional covariates. As above, we then planned to use *stepAIC* to produce a final model that retained only the best predictor variables, based on AIC. We ensured that each model met the relevant assumptions, and we tested for normality using visual inspection of qqplots and the Shapiro test (*p* > 0.05).

#### Relationship between spaceflight changes in PVS characteristics and ventricular volume

We also used stepwise multivariate linear regression to examine whether pre- to post-flight change in ventricular volume predicted changes in PVS characteristics. As there were no differences in ventricular volume changes with flight for the novice versus experienced astronauts, we conducted this analysis across the entire (*n* = 15) astronaut cohort. We first ran a reduced model including ventricular volume as the only predictor. If there was a significant (*p* < 0.05) linear relationship between ventricular volume and the respective PVS metric, we planned to calculate a full model including sex and mean-centered age at launch, total flight duration, and time elapsed from landing to the Return + 4 days MRI scan as additional covariates. Then, as above, we planned to use *stepAIC* to produce a final model that retained only the best predictor variables, based on AIC. We ensured that each model met the relevant assumptions (i.e., independence, outliers, linearity, and normality), and we tested for normality using visual inspection of qqplots and the Shapiro test (*p* > 0.05).

#### Differences in PVS changes for SANS versus no-SANS subgroups

We conducted a final exploratory analysis comparing a subset of astronauts who developed at least one symptom of spaceflight associated neuro-ocular syndrome (SANS, *n* = 6) to the subset of astronauts who did not experience any SANS symptoms (no-SANS, *n* = 6). SANS status was determined using pre- and post-flight ophthalmic exam results obtained from the NASA Lifetime Surveillance of Astronaut Health Repository. We were unable to obtain SANS data for three astronauts, resulting in a sample of 12 for our SANS analysis. As recently defined by Lee et al. 2020^13^ an astronaut was determined to have SANS if they showed changes in the following measures in either eye from pre- to post-flight: optic disc edema (variable Frisén grades), choroidal folds, hyperopic refractive error shifts > 0.75 D, or globe flattening. We conducted identical analyses to those described above (“PVS changes with spaceflight”) to test for pre- to post-flight change in the PVS metrics and ventricular volume, but here we included SANS status as a predictor of interest instead of novice versus experienced status.

## Results

The cohort consisted of 15 astronauts, 9 of whom had no prior flight experience (“novice” astronauts) and 6 of whom had completed at least one previous spaceflight mission (“experienced” astronauts). The experienced astronauts were, on average, older than the novice astronauts. No other novice versus experienced group differences emerged for sex, mission duration, or time between landing and the first post-flight MRI scan (Table 1). The control group consisted of 11 complete cases. There were no group differences in age or sex between the astronauts and controls (Table 1).

### Identification of PVSs and validation of automated PVS algorithm

**Figure 1:**
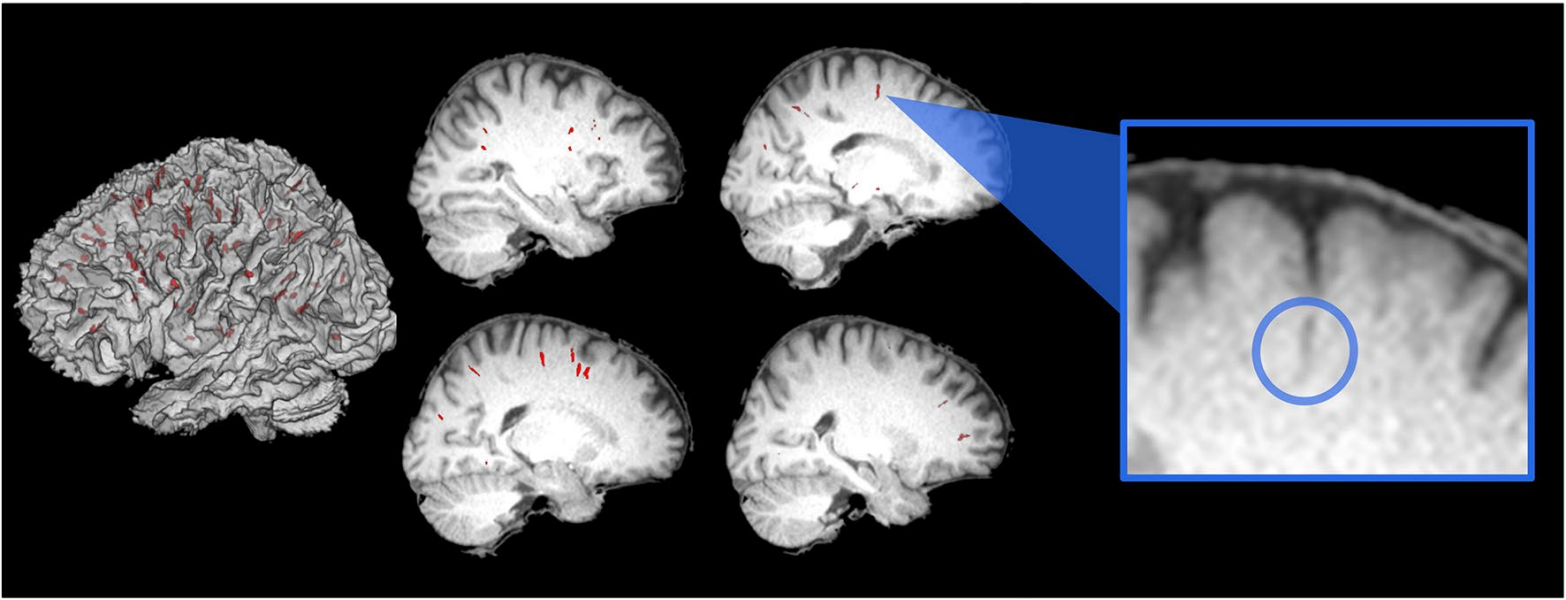
PVSs identified on a single astronaut. Here we depict a binary mask of PVSs for a single astronaut at the last pre-flight time point (Launch-60 days), for illustrative purposes. PVSs are shown in red, overlaid onto this individual’s native space 3D-rendered white matter segment (left) as well as several sagittal slices of their skull stripped native space structural scan (right). The blue box shows a zoomed-in view of one PVS, inside the blue circle.

We identified PVSs in each astronaut and each control at each time point. PVSs were identified in the white matter in various regions across the centrum semiovale white matter (see exemplar participant, Fig. 1) (As described above, our algorithm searched for PVSs only within the white matter, and not within other tissue types).

**Figure 2:**
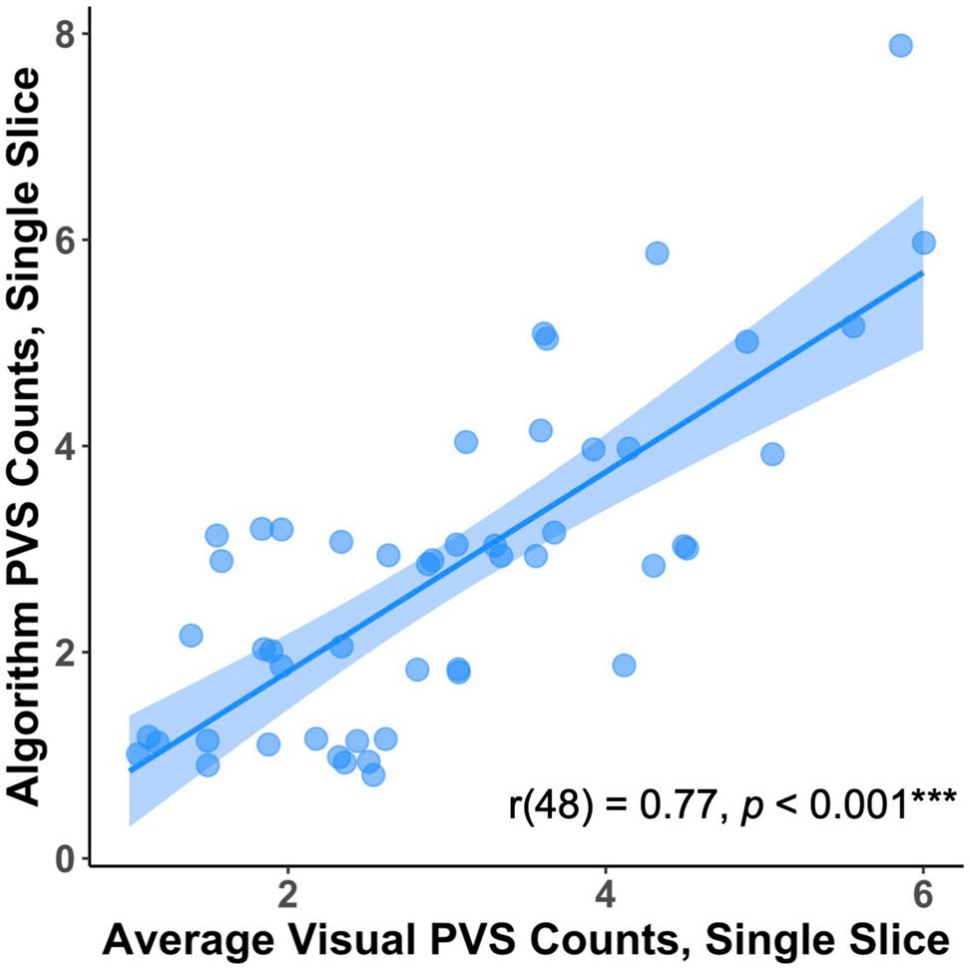
Correlation of total PVS number obtained from automated algorithm and visual rating. Here we depict the strong positive correlation between the algorithm-generated total PVS number, and the average total PVS number (on the same slice for each participant), as counted by two blinded independent raters for 50 randomly selected MRI scans from this astronaut cohort. Note that points are jittered by x = 0.15 and y = 0.15, for ease of visual interpretation (as otherwise, many points would be overlapping with one another).

A Pearson correlation coefficient was computed to assess the linear relationship between total PVS numbers obtained with our automated algorithm and PVS counts obtained by visual ratings (average between two independent, blinded raters). There was a strong positive correlation between the two variables, r(48) = 0.77, *p* < 0.001 (Fig. 2).

### Reliability of PVS metrics

Four of the five PVS metrics, as well as ventricular volume, yielded ICC(3,*k*) values > 0.50 for both the astronauts and the controls. The ICC(3,*k*) values are presented in Table 2.

**Table 2:** Reliability of PVS metrics and ventricular volume.

Measure	ICC(3,*k*) value	
	Astronauts	Controls
Total PVS volume (mm^3^)	0.98	0.99
Total PVS number (No.)	0.97	0.98
Median PVS volume (mm^3^)	0.67	0.79
Median PVS length (mm)	0.59	0.59
Median PVS width (mm)	0.47	0.41
Lateral + third ventricular volume (mL)	1.00	1.00

Here we report the ICC(3,*k*) values across the two pre-flight scans for the astronauts, and across all four scans for the control group. We planned a priori to exclude from further statistical analyses any metric with an ICC(3,*k*) value < 0.50.

We excluded median width from further statistical analyses due to the low reliability of this measure in both the astronauts and controls.

### Changes in PVS characteristics with spaceflight

**Figure 3:**
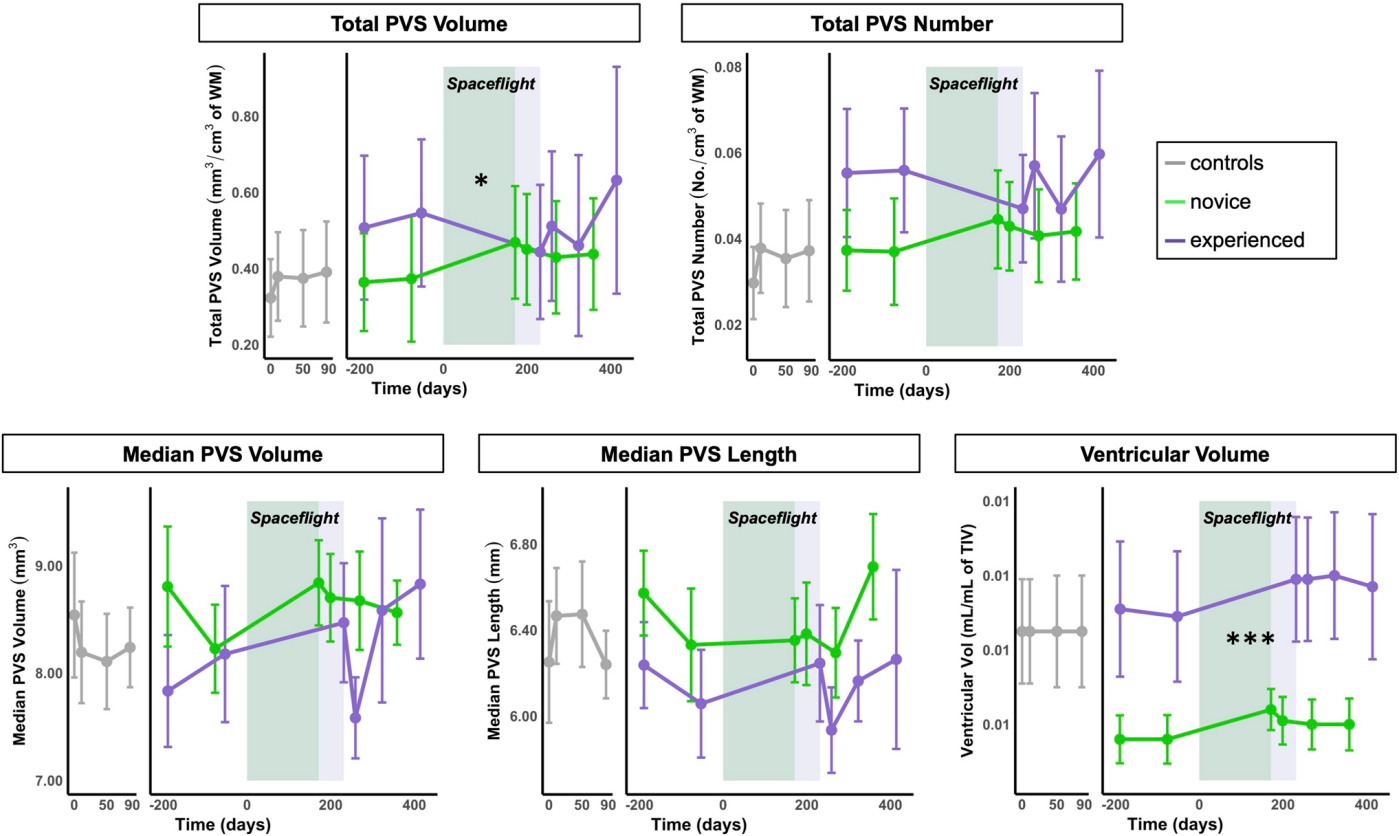
Changes in PVS metrics and ventricular volume from pre- to post-flight. Group average PVS characteristics are depicted for each of the four control group time points (gray, left panels) and for each of the six astronaut time points (right panels). The astronaut data are split into novice (green) and experienced (purple) subgroups. Bars represent standard error. The width of the green and purple boxes indicates the average flight duration for novice and experienced astronauts, respectively. *Indicates a statistically significant (*p* < 0.05) group difference between the novice and experienced astronauts for changes in total PVS volume with spaceflight. ***Indicates a statistically significant (*p* < 0.001) change in ventricular volume from pre- to post-flight. The second astronaut time point depicts group-average data for *n* = 8 (instead of 9) novice astronauts; the final astronaut time point depicts group-average data for *n* = 13 (instead of 15) experienced astronauts (see “Methods” for details on these missing data). We included only complete control group datasets, so *n* = 11 for all control time points. *WM* white matter, *TIV* total intracranial volume.

Changes in PVS characteristics with spaceflight are summarized in Table S1, Table S2, Table 3, and Fig. 3. Table S1 presents PVS and ventricular volume descriptive statistics at each time point. Group mean PVS values reported here fall within the range identified previously by our group for a cohort of male veterans (mean age: 32 years; age range: 29–41 years) with a history of traumatic brain injury^29^. There were no statistically significant whole-group changes in any of the PVS characteristics from pre- to post-flight (*p* > 0.05; Table S2), though, across all astronauts, there was a trend towards pre- to post-flight increases in PVS median length (*p* = 0.069). The final model produced by the stepwise selection procedure for pre- to post-flight changes in total PVS volume included novice versus experienced status as a covariate. The novice astronauts showed a mean pre- to post-flight increase in total PVS volume, whereas the experienced astronauts showed a mean pre- to post-flight decrease in total PVS volume (*p* = 0.020; Table 3; Fig. 3). There was no statistically significant pre-to post-flight difference by novice versus experienced status for the other PVS characteristics, though total PVS number showed a trend (*p* = 0.068) similar to total PVS volume, in which the novice astronauts showed a mean pre- to post-flight increase in total PVS number, whereas the experienced astronauts showed a mean pre- to post-flight decrease in total PVS number. Ventricular volume showed a significant increase from pre- to post-flight for the whole group (*p* < 0.001; Table S2), though there was no effect of experience level on ventricular volume changes (*p* > 0.05; Table 3).

**Table 3:** PVS changes and ventricular expansion from pre- to post-flight: novice vs. experienced differences.

Predictors	Estimates (SE)	95% CI	t	*p*	R^2^/R^2^ adjusted
**Change in total PVS volume (mm**^**3**^**/cm**^**3**^** of WM)**^a^					
(*Intercept*)	− 0.10 (0.05)	− 0.20 to 0.01	− 2.00	0.067	
Flight experience (*Novice*)	0.16 (0.06)	0.03 to 0.30	2.65	0.020*	
					0.35/0.30
**Change in total PVS number (No./cm**^**3**^** of WM)**^a^					
(*Intercept*)	− 0.01 (0.01)	− 0.02 to 0.003	− 1.65	0.123	
Flight experience (*Novice*)	0.01 (0.01)	− 0.001 to 0.03	1.99	0.068	
					0.23/0.18
**Change in median PVS volume (mm**^**3**^**)**					
(*Intercept*)	0.29 (0.45)	− 0.68 to 1.26	0.65	0.527	
Flight experience (*Novice*)	0.43 (0.58)	− 0.82 to 1.68	0.74	0.471	
					0.04/− 0.03
**Change in median PVS length (mm)**					
(*Intercept*)	0.19 (0.22)	− 0.28 to 0.66	0.87	0.401	
Flight experience (*Novice*)	− 0.20 (0.28)	− 0.81 to 0.40	− 0.72	0.484	
					0.04/− 0.04
**Change in ventricular volume (mL/mL of TIV)**^b^					
(*Intercept*)	0.001 (0.0002)	0.001 to 0.001	6.16	0.002**	
Flight experience (*Novice*)	− 0.0002 (0.0002)	− 0.001 to 0.0003	− 0.97	0.349	
					0.07/− 0.004

*p < 0.05, **p < 0.01; significant *p* values are bolded. *SE* standard error, *CI* confidence interval, *WM *white matter, *TIV* total intracranial volume. Here we report the results of linear models testing whether the pre- to post-flight change in each PVS metric and ventricular volume differed for the novice vs. experienced astronauts. Experienced astronauts served as the reference group. Our primary interest here was whether there was an effect of previous flight exposure on pre- to post-flight change in the PVS and ventricle metrics. Note that the novice vs. experienced predictor was the only variable that the stepwise regression included in the final statistical model for total PVS volume. Age, sex, time elapsed between landing and first MRI scan, and flight duration were not included in the final model produced by the stepwise approach. See Table S1 for whole-group and subgroup descriptive statistics at each time point. See Table S2 for the results of the model testing for pre- to post-flight changes in PVS metrics and ventricular volume for the entire (*n* = 15) astronaut cohort.

^a^To account for individual differences in total brain tissue volumes, total PVS volume and number were normalized as follows: (total PVS volume (mm^3^) or number (No.))/(total brain white matter volume (cm^3^), averaged across the two pre-flight time points).

^b^Ventricular volume represents the sum of the lateral and third ventricular volumes. To account for individual differences in head size, ventricular volume was then normalized as follows: (ventricular volume (mL))/(total intracranial volume (mL), averaged across the two pre-flight time points).

### Correlation between past flight experience and baseline PVS characteristics

The correlations between past flight experience and baseline PVS characteristics are summarized in Table S3. Among the experienced astronauts (*n* = 6), there were no statistically significant relationships between past flight experience and baseline PVS characteristics (*p* > 0.05). However, each relationship was of large effect size (r = 0.60–0.71) and indicated that a greater number of previous flight days was associated with greater pre-flight PVS total volume (r(4) = 0.61; *p* = 0.201), total number (r(4) = 0.60; *p* = 0.208), median volume (r(4) = 0.71; *p* = 0.114), and median length (r(4) = 0.70; *p* = 0.121). These strong effect sizes remained similar when also controlling for age at launch (partial r = 0.69 to 0.77). There was no correlation between number of previous flight days and ventricular volume (r(4) = −0.08; *p* = 0.873).

### Correlation between post-flight ventricular expansion and PVS characteristics

The correlations between post-flight ventricular expansion and PVS characteristics are summarized in Table S4. There was one relationship of moderate strength between greater pre- to post-flight change in ventricular volume and greater pre- to post-flight change in median PVS length, though this did not reach statistical significance (r(13) = 0.44; *p* = 0.100). There was no statistically significant correlation between ventricular volume changes and the other PVS characteristics (r = − 0.12 to − 0.25; *p* > 0.05).

### No group differences by SANS status

There were no statistically significant changes in PVS metrics or ventricular volume at baseline from pre- to post-flight between those who did and did not experience one or more symptom(s) of SANS (Table S5).

## Discussion

This is the first study to examine changes in MRI-visible PVS in the white matter with spaceflight, and to demonstrate that PVS metrics can be consistently identified on *T*_1_-weighted MRI scans using an automated algorithm. Our key findings included that novice astronauts exhibited increases in total PVS volume from pre- to post-flight, whereas experienced astronauts did not. Moreover, in experienced astronauts, the number of days spent in space prior to the current mission was associated with greater PVS load at the two pre-flight scan time points, though these relationships did not reach statistical significance. We also identified a moderate correlation between pre- to post-flight increases in PVS median length and pre- to post-flight increases in the sum of lateral and third ventricular volume, though this relationship also did not reach statistical significance. Finally, we did not find any significant differences in PVS characteristics between those who did and did not exhibit one or more symptom(s) of SANS.

We found that novice astronauts exhibited an increase in PVS volume from pre- to post-flight, whereas experienced flyers did not. This divergence between novice and experienced astronauts is consistent with the hypothesis that ventricular expansion with spaceflight is a compensatory response^49^. That is, Roberts and colleagues suggest that experienced flyers have larger pre-flight ventricular volume (i.e., lingering ventricular expansion from previous flights^5,6,10,11^), and therefore do not show as much ventricular volume increase with subsequent spaceflights, perhaps due to a loss of compliance with changes from previous flights. Whether the PVS findings observed here reflect pathophysiological or compensatory consequences of spaceflight remains unknown. Regardless, repeated spaceflight exposure appears to be a key determinant of brain fluid changes that should be further explored in the future. That is, these data suggest a cumulative and asymptotic effect on CSF spaces. As astronauts experience microgravity for longer cumulative durations, ventricles enlarge, and PVS size increases, eventually reaching a plateau, which potentially decreases the changes observed with each subsequent flight.

In line with this hypothesis, we also found that, among the experienced crewmembers, greater PVS load (i.e., total volume, total number, median volume, and median length) at baseline was correlated with more days previously spent in space (though these relationships did not reach statistical significance and should be interpreted with caution). This fits with previous work from our group and others, showing that alterations in gray matter^4,5^, white matter microstructure^8^, *T*_2_ hyperintensities^12^, free water distribution^8^, and ventricular volume^3,5,9,12^ depend on the frequency and/or duration of time spent in space. This finding also suggests that PVS changes with spaceflight may be long lasting. Indeed, previous spaceflight longitudinal analyses have suggested that several structural brain changes with flight (namely, ventricular expansion) are slow to recover, remaining elevated at 6 to 12 months post-flight for some individuals^5,6,10,11^. Therefore, as NASA’s goals shift towards longer-duration missions to Mars, it is becoming increasingly critical to study the mechanisms and consequences of PVS enlargement in future spaceflight or ground-based analog studies, ideally with larger samples.

Furthermore, we observed a moderate positive correlation between greater increase in ventricular volume with flight and greater increase in median PVS length (though this relationship did not reach statistical significance and thus we hesitate to over-interpret this result). However, it is worth noting that multiple previous studies have shown cranial fluid and brain position shifts following spaceflight^3,5,6,8^, and these shifts could compress the adjacent venous structures at the top of the head. This could then obstruct the arachnoid granulations positioned along the superior sagittal sinus and/or compress the dural lymphatic vessels, further impairing CSF reabsorption. Roberts et al.^49^ have suggested that the result of this CSF outflow obstruction is ventricular dilation similar to that observed in individuals with normal pressure hydrocephalus. Under this assumption, the moderate relationship between pre- to post-flight changes in PVS length and ventricular expansion could represent the result of CSF resorption obstruction in light of a fundamental venous cranial outflow obstruction due to cephalad fluid shift. It is worth noting that individuals with normal pressure hydrocephalus show both dilated PVS and glymphatic dysfunction represented by delayed clearance of intrathecally administered gadolinium contrast^50–52^.

In our cohort, we did not observe an association between changes in PVS characteristics and clinical manifestations of SANS. Headward fluid shifts leading to obstruction of CSF and intraorbital fluid and glymphatic dysfunction have been proposed as potential mechanisms underlying SANS^13^. There are several plausible explanations for the lack of relationship between PVS change and SANS symptoms. First, it may be that PVS function does not relate to SANS. Second, the role of PVS in CSF clearance remains unknown, and it is possible that impaired clearance still occurs in the absence of PVS enlargement^19^. Third, the absence of correlations between PVS and SANS symptoms could be a consequence of our small subgroup sizes.

Factors aside from long-duration microgravity exposure alone may also influence spaceflight effects on PVS. For instance, as suggested by evidence in subjects with traumatic brain injuries^23,29^, external mechanical forces (i.e., the high accelerations that occur with launch and landing) may contribute to PVS changes in astronauts. However, our recent case study in a single astronaut^53^ suggests that structural brain changes with spaceflight (i.e., fluid shifts within the brain and ventricular expansion) are more likely to be due to microgravity than to the accelerations of launch/landing (though we did not examine PVS in that study). Moreover, normal aging could influence longitudinal PVS changes. However, the pre-flight stability of the PVS metrics (over about 4 months), as well as the stability of the PVS metrics in the control group (over about 3 months) suggests that normal aging in our middle-aged astronauts did not substantially influence the observed longitudinal PVS changes. In addition, in the one case where we observed differences between novice and experienced astronauts, the older mean age of the experienced astronaut group likely did not affect these group differences. We showed that PVS total volume *increased* for the novice astronauts but *decreased* for the experienced astronauts from pre- to post-flight. This would be the opposite direction of what we would expect if we were only capturing normal aging changes in PVS metrics.

Parallel to what occurs with neurodegenerative disease (e.g., Alzheimer’s disease), PVS blockage and enlargement might suggest an inability to clear harmful metabolic waste (e.g., amyloid) from the draining pathway, leading to an accumulation of neurotoxic protein aggregates around the blood vessel^54^. This buildup is believed to result in a disruption to brain homeostasis and is associated with the pathogenesis of Alzheimer’s disease; however, the specific mechanisms driving PVS enlargement are unknown^55^. Recently in five cosmonauts, blood markers for amyloid-β (Aβ_40_ and Aβ_42_) proteins frequently linked with neurodegenerative disease and brain injury were identified to increase from pre- to post-spaceflight and remained elevated three weeks after return^56^. This post-flight amyloid-β elevation was described as a potential reparatory process, acting to restore the integrity of the blood–brain barrier^56^; the potential role of PVS clearance in this maintenance process should be examined in future spaceflight studies.

Our study has several limitations. Our original study design, developed over a decade ago, did not aim to examine PVS changes with spaceflight. However, recent reports of PVS changes in aging^30,57^, brain injury^25,29^, and neurologic disease populations^19,20,22,55,58^ led us to hypothesize that spaceflight might also cause changes in PVS number and morphology. Moreover, the number of people who travel to space is small, creating an inevitable obstacle in the efforts to study spaceflight-associated changes. To address the concern of sample size, a repeated measures design was implemented, thereby allowing each astronaut to serve as their own control. Given this limited sample size, the statistical analyses (and particularly, correlation results) should be interpreted with caution and replicated in future studies as more individuals travel to space.

Though we included a ground-based control group for comparison purposes, this control group was originally intended for use with multiple spaceflight and analog studies. As a result, the testing timeline and demographics for this control group were not specifically matched to the current study. Thus, here we leveraged the control group only to provide additional evidence that PVS metrics are stable over time when participants go about their daily lives on Earth. An additional limitation is that we did not have access to phantom data to perform our own quality controls on the data; however, the scans were all collected at the same, active clinical MRI facility, which regularly performs its own quality controls. There was also a delay between re-entry to Earth's gravitational field and the first post-flight MRI acquisition, ranging between one and six days after landing (average = 4.5 days). We used this value as a covariate in the full statistical models to mitigate its effect. Given that ventricular volume changes are still evident months post-flight^5,6,10,11^, this time offset is likely not a key factor in the current study. However, it could be that PVS and ventricular volume changes have different temporal dynamics and recovery patterns.

Another limitation is that the PVS algorithm we employed is tailored to white matter PVS segmentation and has not yet been optimized for identifying PVS in the gray matter; future studies should enhance the algorithm to incorporate other tissue types and anatomical regions. For example, future studies planning to characterize PVS changes with spaceflight should optimize acquisition protocols for PVS detection across the whole brain (e.g., by collecting structural images with isotropic voxels, instead of resampling to isotropic voxel sizes after acquisition^59^). Moreover, it is possible that the PVS segmentation algorithm is not sensitive enough to uncover subtle differences in PVS characteristics caused by spaceflight. However, despite large between-subject differences, ICCs between the two pre-flight MRIs and among the control participants met our a priori inclusion criteria for all but one (i.e., median width) PVS metric. Due to the resolution of the images available, we were not able to distinguish whether the quantified PVSs were arterial or venular. Moreover, although it is presumed that we identified MRI-visible PVSs, no quantitative delineation was instituted to rule out the presence of neuroanatomical anomalies (i.e., *T*_1_ hypointensities, small vessel disease, etc.). Lastly, while our algorithm is able to detect PVS in *T*_1_-weighted images, the absence of a *T*_2_-weighted scan prevents PVS from being compared with *T*_2_ white matter hyperintensities (i.e., *T*_1_ hypointensities). Despite finding a positive correlation between our PVS detection algorithm with the results of two experienced raters, future studies aimed at characterizing PVS changes with spaceflight will benefit from a more comprehensive imaging acquisition protocol that includes *T*_2_-weighted FLAIR sequences. The use of additional sequences such as *T*_2_ to confirm that findings on *T*_1_ are indeed PVS remains the preferred method for PVS characterization, particularly in older individuals. However, given that our sample comprises very healthy individuals, with a mean age in their mid-40s, we do not anticipate that *T*_2_ white matter hyperintensities posed a large problem for PVS identification in this cohort. For example, in a cohort of individuals ages 16–65 years, Hopkins et al.^60^ found that *T*_2_ white matter hyperintensities occurred in only 13 of these 243 individuals (i.e., 5.3% of the cohort). Moreover, those older than 55 years had a tenfold increase in hyperintensity prevalence compared to those ≤ 55 years. Thus, in this healthy astronaut cohort, we do not anticipate that *T*_2_ white matter hyperintensities were a substantial confounding factor in relation to the PVS metrics.

## Conclusion

We found that at the group level, PVS was not affected by spaceflight. However, when we evaluated these changes separately between novice astronauts and those that had been to space before, we observed experience-dependent effects. First, novice astronauts had an increase in PVS with spaceflight, whereas experienced crewmembers did not. Second, those with prior spaceflight experience exhibited a correlation between more previous days in space and greater pre-flight PVS length (though this effect did not reach statistical significance). Our findings suggest that PVS changes with spaceflight are dependent upon prior microgravity exposure, similar to other brain structural changes we have reported^5^. Whether these changes in PVS represent a compensatory mechanism or could eventually lead to pathological manifestations remains unknown. Given the moderate (though not statistically significant) relationship we identified between greater increases in ventricular volume with flight and greater increases in PVS length, we suggest that our findings relate to CSF resorption obstruction caused by the fluid^3,6,8^ and upward brain position shifts^3,5,6,8^ observed with microgravity exposure. Moving forward, head-down tilt bed rest analog studies may provide further insights into PVS associations with spaceflight, as this environment also results in brain position and fluid shifts.

## Supplementary Information

Supplementary Tables.

## Data Availability

The datasets included in this study will be made available through NASA’s Life Sciences Data Archive (https://lsda.jsc.nasa.gov), upon reasonable request and with permission of NASA.
