# Supplementary material for: Longitudinal MRI-visible perivascular space (PVS) changes with long-duration spaceflight
Source: Sci Rep. 2022 May 5;12:7238. doi: 10.1038/s41598-022-11593-y (PMC9072425; doi:10.1038/s41598-022-11593-y)
Supplement: Supplementary file 1 — Supplementary Tables. [file 41598_2022_11593_MOESM1_ESM.pdf]

Supplementary Information:

Longitudinal MRI-Visible Perivascular Space (PVS) Changes with Long-Duration Spaceflight

Kathleen E. Hupfeld[a†], Sutton B. Richmond[a†], Heather R. McGregor[a],
Daniel L. Schwartz[b,c], Madison Luther[d], Nichole E. Beltran[e], Igor S. Kofman[e], Yiri E. De Dios[e],
Roy F. Riascos[f], Scott J. Wood[g], Jacob J. Bloomberg[h], Ajitkumar P. Mulavara[e], Lisa C. Silbert[b,i],
Jeffrey J. Iliff[j,k,l], Rachael D. Seidler[a,m], and Juan Piantino[d*]

[†]denoting co-first authorship

[a] Department of Applied Physiology and Kinesiology, University of Florida, 1864 Stadium Rd., Gainesville, FL, USA

[b] Layton-NIA Oregon Aging and Alzheimer's Disease Research Center, Department of Neurology, Oregon Health & Science University, Portland, OR, USA

[c] Advanced Imaging Research Center, Oregon Health & Science University, Portland, OR, USA

[d] Department of Pediatrics, Division of Child Neurology, Doernbecher Children's Hospital, Oregon Health and Science University, Portland, OR, USA

[e] KBR, Houston, TX, USA

[f] Department of Diagnostic and Interventional Imaging, University of Texas Health Science Center at Houston, Houston, TX, USA

[g] NASA Johnson Space Center, Houston, TX, USA

[h] NASA Johnson Space Center, *retired*, Houston, TX, USA

[i] Veteran's Affairs Portland Health Care System, Neurology, Portland, OR, USA

[j] Department of Psychiatry and Behavioral Sciences, University of Washington School of Medicine, Seattle, WA, USA

[k] Department of Neurology, University of Washington School of Medicine, Seattle, WA, USA

[l] VISN 20 Mental Illness Research, Education and Clinical Center (MIRECC), VA Puget Sound Health Care System, Seattle, WA, USA

[m] Norman Fixel Institute for Neurological Diseases, University of Florida, Gainesville, FL, USA

Table S1. PVS Metrics at Each Time Point for the Whole Astronaut Cohort and Subgroups

	**All Astronauts (*n* = 15)**		**Experienced (*n* = 6)**		**Novice (*n* = 9)**	
**Time Point**	**Mean (SD)**	**95% CI**	**Mean (SD)**	**95% CI**	**Mean (SD)**	**95% CI**
**Total PVS Volume (mm^3/cm^3 of WM)[a]**						
Scan 1: Pre-Flight, Launch – 180 days	0.46 (0.38)	0.25 - 0.67	0.54 (0.43)	0.08 - 0.99	0.40 (0.36)	0.13 - 0.68
Scan 2: Pre-Flight, Launch – 60 days	0.48 (0.43)	0.23 - 0.73	0.57 (0.44)	0.11 - 1.04	0.41 (0.44)	0.05 - 0.78
Scan 3: Post-Flight, Return + 4 days	0.49 (0.39)	0.27 - 0.71	0.48 (0.40)	0.05 - 0.90	0.50 (0.41)	0.18 - 0.82
Scan 4: Pre-Flight, Return + 30 days	0.51 (0.41)	0.28 - 0.73	0.54 (0.45)	0.07 - 1.01	0.48 (0.41)	0.17 - 0.80
Scan 5: Pre-Flight, Return + 90 days	0.51 (0.45)	0.25 - 0.77	0.49 (0.54)	-0.08 - 1.06	0.52 (0.41)	0.18 - 0.86
Scan 6: Pre-Flight, Return + 180 days	0.53 (0.44)	0.26 - 0.79	0.65 (0.55)	-0.23 - 1.54	0.47 (0.41)	0.16 - 0.79
**Total PVS Number (No./cm^3 of WM)[a]**						
Scan 1: Pre-Flight, Launch – 180 days	4.45x10^{-2} (3.18x10^{-2})	2.68x10^{-2} - 6.21x10^{-2}	5.53x10^{-2} (3.64x10^{-2})	1.71x10^{-2} - 9.35x10^{-2}	3.73x10^{-2} (2.82x10^{-2})	1.56x10^{-2} - 5.90x10^{-2}
Scan 2: Pre-Flight, Launch – 60 days	4.51x10^{-2} (3.52x10^{-2})	2.47x10^{-2} - 6.54x10^{-2}	5.59x10^{-2} (3.53x10^{-2})	1.88x10^{-2} - 9.30x10^{-2}	3.70x10^{-2} (3.52x10^{-2})	0.76x10^{-2} - 6.64x10^{-2}
Scan 3: Post-Flight, Return + 4 days	4.55x10^{-2} (3.16x10^{-2})	2.80x10^{-2} - 6.30x10^{-2}	4.70x10^{-2} (3.06x10^{-2})	1.49x10^{-2} - 7.91x10^{-2}	4.45x10^{-2} (3.41x10^{-2})	1.83x10^{-2} - 7.07x10^{-2}
Scan 4: Pre-Flight, Return + 30 days	4.86x10^{-2} (3.48x10^{-2})	2.93x10^{-2} - 6.78x10^{-2}	5.70x10^{-2} (4.14x10^{-2})	1.36x10^{-2} - 10.05x10^{-2}	4.29x10^{-2} (3.08x10^{-2})	1.92x10^{-2} - 6.66x10^{-2}
Scan 5: Pre-Flight, Return + 90 days	4.60x10^{-2} (3.45x10^{-2})	2.60x10^{-2} - 6.59x10^{-2}	4.69x10^{-2} (4.15x10^{-2})	0.33x10^{-2} - 9.04x10^{-2}	4.53x10^{-2} (3.13x10^{-2})	1.91x10^{-2} - 7.14x10^{-2}
Scan 6: Pre-Flight, Return + 180 days	4.73x10^{-2} (3.47x10^{-2})	2.63x10^{-2} - 6.82x10^{-2}	5.97x10^{-2} (3.87x10^{-2})	-0.20x10^{-2} - 12.13x10^{-2}	4.17x10^{-2} (3.36x10^{-2})	1.59x10^{-2} - 6.76x10^{-2}
**Median PVS Volume (mm^3)**						
Scan 1: Pre-Flight, Launch – 180 days	8.42 (1.56)	7.55 - 9.28	7.83 (1.28)	6.49 - 9.17	8.81 (1.68)	7.52 - 10.09
Scan 2: Pre-Flight, Launch – 60 days	8.21 (1.28)	7.46 - 8.95	8.18 (1.55)	6.55 - 9.81	8.23 (1.16)	7.26 - 9.20
Scan 3: Post-Flight, Return + 4 days	8.69 (1.23)	8.01 - 9.37	8.47 (1.36)	7.04 - 9.89	8.84 (1.19)	7.93 - 9.75
Scan 4: Pre-Flight, Return + 30 days	8.25 (1.22)	7.58 - 8.93	7.58 (0.92)	6.61 - 8.55	8.70 (1.22)	7.76 - 9.64
Scan 5: Pre-Flight, Return + 90 days	8.51 (1.61)	7.58 - 9.44	8.58 (2.10)	6.38 - 10.79	8.45 (1.29)	7.38 - 9.53
Scan 6: Pre-Flight, Return + 180 days	8.64 (1.02)	8.03 - 9.26	8.83 (1.39)	6.62 - 11.04	8.56 (0.90)	7.87 - 9.25
**Median PVS Length (mm)**						
Scan 1: Pre-Flight, Launch – 180 days	6.44 (0.56)	6.13 - 6.75	6.24 (0.49)	5.72 - 6.75	6.57 (0.59)	6.12 - 7.03
Scan 2: Pre-Flight, Launch – 60 days	6.21 (0.68)	5.82 - 6.61	6.06 (0.61)	5.41 - 6.70	6.33 (0.74)	5.71 - 6.95
Scan 3: Post-Flight, Return + 4 days	6.31 (0.60)	5.98 - 6.64	6.25 (0.66)	5.55 - 6.94	6.35 (0.59)	5.90 - 6.80
Scan 4: Pre-Flight, Return + 30 days	6.20 (0.66)	5.84 - 6.57	5.93 (0.49)	5.42 - 6.45	6.38 (0.72)	5.83 - 6.93
Scan 5: Pre-Flight, Return + 90 days	6.30 (0.53)	5.99 - 6.60	6.16 (0.46)	5.68 - 6.65	6.40 (0.58)	5.91 - 6.88
Scan 6: Pre-Flight, Return + 180 days	6.56 (0.76)	6.10 - 7.02	6.26 (0.83)	4.94 - 7.59	6.70 (0.74)	6.13 - 7.26
**Ventricular Volume (mL/mL of TIV)[b]**						
Scan 1: Pre-Flight, Launch – 180 days	9.00x10^{-3} (3.52x10^{-3})	7.05x10^{-3} - 10.95x10^{-3}	11.12x10^{-3} (4.45x10^{-3})	6.46x10^{-3} - 15.79x10^{-3}	7.59x10^{-3} (1.93x10^{-3})	6.11x10^{-3} - 9.07x10^{-3}
Scan 2: Pre-Flight, Launch – 60 days	8.90x10^{-3} (3.41x10^{-3})	7.01x10^{-3} - 10.78x10^{-3}	10.91x10^{-3} (4.29x10^{-3})	6.40x10^{-3} - 15.41x10^{-3}	7.56x10^{-3} (1.95x10^{-3})	6.05x10^{-3} - 9.06x10^{-3}
Scan 3: Post-Flight, Return + 4 days	9.80x10^{-3} (3.29x10^{-3})	7.98x10^{-3} - 11.62x10^{-3}	11.94x10^{-3} (4.10x10^{-3})	7.64x10^{-3} - 16.24x10^{-3}	8.38x10^{-3} (1.66x10^{-3})	7.10x10^{-3} - 9.65x10^{-3}
Scan 4: Pre-Flight, Return + 30 days	9.62x10^{-3} (3.40x10^{-3})	7.73x10^{-3} - 11.51x10^{-3}	11.86x10^{-3} (4.06x10^{-3})	8.13x10^{-3} - 1.92x10^{-3}	8.13x10^{-3} (1.92x10^{-3})	6.65x10^{-3} - 9.60x10^{-3}
Scan 5: Pre-Flight, Return + 90 days	9.88x10^{-3} (3.53x10^{-3})	7.84x10^{-3} - 11.92x10^{-3}	12.02x10^{-3} (4.16x10^{-3})	7.65x10^{-3} - 16.39x10^{-3}	8.27x10^{-3} (1.98x10^{-3})	6.61x10^{-3} - 9.93x10^{-3}

Scan 6: Pre-Flight, Return + 180 days	$9.35x10^{-3}$ ($3.46x10^{-3}$)	$7.35x10^{-3}$ - $11.35x10^{-3}$	$11.74x10^{-3}$ ($4.36x10^{-3}$)	$6.33x10^{-3}$ - $17.15x10^{-3}$	$8.02x10^{-3}$ ($2.09x10^{-3}$)	$6.42x10^{-3}$ - $9.63x10^{-3}$

Table S1 Note. SD = standard deviation, CI = confidence interval, WM = white matter, TIV = total intracranial volume. Here we report descriptive statistics for each PVS metric and ventricular volume at all of the testing time points, for the whole astronaut cohort and for the experienced and novice subgroups.

[a] To account for individual differences in total brain tissue volumes, total PVS volume and number were normalized as follows: (total PVS volume (mm^3) or number (No.)) / (total brain white matter volume (cm^3), averaged across the two pre-flight time points).

[b] Ventricular volume represents the sum of the lateral and third ventricular volumes. To account for individual differences in head size, ventricular volume was then normalized as follows: (ventricular volume (mL)) / (total intracranial volume (mL), averaged across the two pre-flight time points).

Table S2. PVS Changes and Ventricular Expansion from Pre- to Post-Flight

Predictors	Estimates (SE)	95% CI	t	*p*
**Total PVS Volume (mm^3/cm^3 of WM)[a]**				
(Intercept)	0.003 (0.04)	-0.07 - 0.08	0.07	0.944
**Total PVS Number (No./cm^3 of WM)[a]**				
(Intercept)	-0.001 (0.004)	-0.01 - 0.01	-0.16	0.879
**Median PVS Volume (mm^3)**				
(Intercept)	0.55 (0.28)	-0.05 - 1.15	1.97	0.069
**Median PVS Length (mm)**				
(Intercept)	0.07 (0.13)	-0.22 - 0.36	0.50	0.626
**Ventricular Volume (mL/mL of TIV)[b]**				
(Intercept)	0.001 (0.0001)	0.001 - 0.001	8.57	**< 0.001*** **

Table S2 Note. ***$p < 0.001$; significant *p* values are bolded. SE = standard error, CI = confidence interval, WM = white matter, TIV = total intracranial volume. Here we report the results of linear models testing whether the change in each PVS metric and ventricular volume from pre- to post-flight differed significantly from 0. Our primary interest here was whether the intercept was significant ($p < 0.05$), thereby indicating a significant whole-group change in the PVS metric or ventricular volume with spaceflight.

[a] To account for individual differences in total brain tissue volumes, total PVS volume and number were normalized as follows: (total PVS volume (mm^3) or number (No.)) / (total brain white matter volume (cm^3), averaged across the two pre-flight time points).

[b] Ventricular volume represents the sum of the lateral and third ventricular volumes. To account for individual differences in head size, ventricular volume was then normalized as follows: (ventricular volume (mL)) / (total intracranial volume (mL), averaged across the two pre-flight time points).

Table S3. Correlation of Pre-Flight PVS Characteristics and Ventricular Volume with Number of Past Flight Days

Predictors	Pearson r	*p*
Pre-Flight Total PVS Volume (mm^3/cm^3 of WM)[a]	0.61	0.201
Pre-Flight Total PVS Number (No./cm^3 of WM)[a]	0.60	0.208
Pre-Flight Median PVS Volume (mm^3)	0.71	0.114
Pre-Flight Median PVS Length (mm)	0.70	0.121
Pre-Flight Ventricular Volume (mL/mL of TIV)[b]	-0.08	0.873

Table S3 Note. WM = white matter, TIV = total intracranial volume. Here we report the results of Pearson correlation tests for relationships between total number of past flight days and average baseline PVS and ventricular volume metrics (i.e., across the two pre-flight time points) for the experienced astronauts only (n = 6, degrees of freedom = 4).

[a] To account for individual differences in total brain tissue volumes, total PVS volume and number at baseline were normalized as follows: (total PVS volume (mm^3) or number (No.), averaged across the two pre-flight time points) / (total brain white matter volume (cm^3), averaged across the two pre-flight time points).

[b] Ventricular volume represents the sum of the lateral and third ventricular volumes. To account for individual differences in head size, ventricular volume at baseline was then normalized as follows: (ventricular volume (mL), averaged across the two pre-flight time points) / (total intracranial volume (mL), averaged across the two pre-flight time points).

Table S4. Correlation Between Pre- to Post-Flight Ventricular Expansion and Pre- to Post-Flight Changes in PVS Characteristics

Predictors	Pearson r (DF)	*p*
Change in Total PVS Volume (mm^3/cm^3 of WM)[a]	-0.25	0.367
Pre-Flight Total PVS Number (No./cm^3 of WM)[a]	-0.26	0.353
Pre-Flight Median PVS Volume (mm^3)	-0.12	0.659
Pre-Flight Median PVS Length (mm)	0.44	0.100

Table S4 Note. $*p < 0.05$; significant *p* values are bolded. WM = white matter. Here we report the results of Pearson correlation tests for relationships between change in PVS metrics from pre- to post-flight and change in ventricular volume from pre- to post-flight for the entire astronaut cohort ($n = 15$, degrees of freedom = 13).

[a] To account for individual differences in total brain tissue volumes, total PVS volume and number were normalized as follows: (total PVS volume (mm^3) or number (No.)) / (total brain white matter volume (cm^3), averaged across the two pre-flight time points).

[b] Ventricular volume represents the sum of the lateral and third ventricular volumes. To account for individual differences in head size, ventricular volume was then normalized as follows: (ventricular volume (mL)) / (total intracranial volume (mL), averaged across the two pre-flight time points).

Table S5. PVS Changes and Ventricular Expansion from Pre- to Post-Flight: SANS vs. no-SANS Differences

Predictors	Estimates (SE)	95% CI	t	*p*	R^2 / R^2 Adjusted
**Change in Total PVS Volume (mm^3/cm^3 of WM)[a]**					
(Intercept)	0.05 (0.05)	-0.07 - 0.17	0.86	0.410	
SANS Status *(SANS)*	-0.04 (0.08)	-0.21 - 0.13	-0.52	0.616	
					0.03 / -0.07
**Change in Total PVS Number (No./cm^3 of WM)[a]**					
(Intercept)	0.002 (0.01)	-0.01 - 0.02	0.48	0.641	
SANS Status *(SANS)*	-0.002 (0.01)	-0.02 - 0.02	-0.29	0.775	
					0.01 / -0.09
**Change in Median PVS Volume (mm^3)**					
(Intercept)	0.50 (0.48)	-0.56 - 1.56	1.05	0.319	
SANS Status *(SANS)*	-0.22 (0.67)	-1.72 - 1.28	-0.33	0.752	
					0.01 / -0.09
**Change in Median PVS Length (mm)**					
(Intercept)	-0.03 (0.24)	-0.57 - 0.50	-0.14	0.890	
SANS Status *(SANS)*	0.10 (0.34)	-0.66 - 0.86	0.30	0.768	
					0.01 / -0.09
**Change in Ventricular Volume (mL/mL of TIV)[b]**					
(Intercept)	0.001 (0.0002)	0.0003 - 0.001	4.22	**0.002****	
SANS Status *(SANS)*	0.0002 (0.0002)	-0.0003 - 0.001	1.02	0.331	
					0.10 / 0.004

Table S5 Note. SE = standard error, CI = confidence interval, WM = white matter, TIV = total intracranial volume. Here we report the results of linear models testing whether the pre- to post-flight change in each PVS metric and ventricular volume differed for the SANS vs. no-SANS astronauts. No-SANS served as the reference group (i.e., coded as = 0). Our primary interest here was whether there was an effect of SANS status on pre- to post-flight change in the PVS and ventricle metrics.

[a] To account for individual differences in total brain tissue volumes, total PVS volume and number were normalized as follows: (total PVS volume (mm^3) or number (No.)) / (total brain white matter volume (cm^3), averaged across the two pre-flight time points).

[b] Ventricular volume represents the sum of the lateral and third ventricular volumes. To account for individual differences in head size, ventricular volume was then normalized as follows: (ventricular volume (mL)) / (total intracranial volume (mL), averaged across the two pre-flight time points).
